# Supplementary material for: Benchmarking spatial transcriptomics technologies with the multi-sample SpatialBenchVisium dataset
Source: Genome Biol. 2025 Mar 28;26:77. doi: 10.1186/s13059-025-03543-4 (PMC11954323; doi:10.1186/s13059-025-03543-4)
Supplement: Supplementary file 2 — Additional file 2: Fig. S1, Fig. S2, Fig. S3, Fig. S4, Fig. S5, Fig. S6, Fig. S7, Fig. S8, Fig. S9, Fig. S10, Fig. S11, Fig. S12, Fig. S13, Fig. S14, Fig. S15. [file 13059_2025_3543_MOESM2_ESM.pdf]

Contents

Supplementary Tables

S1 Summary of experiments. 2
S2 Summary of quality control metrics. 2
S3 Marker gene list used to annotate spatial clusters as different cell types or tissue regions in the mouse spleen 3

Supplementary Figures

S1 UMI count spatial distribution plots. 4
S2 Upset plot showing the overlap of detected genes in all wildtype samples. 5
S3 MDS plot of pseudo-bulk counts from all samples 6
S4 Variable gene (SVGs and HVGs) analyses. 7
S5 Spatial expression of the top SVGs in FFPE CA samples. 8
S6 Spleen structure segmentation in FFPE CA samples with a trained classifier. 9
S7 UMAP plot of FFPE CA sample group following batch correction. 10
S8 Cell type annotation and composition across all protocols. 11
S9 Erythrocyte classification following deconvolution of FFPE CA samples. 12
S10 Spatial plot of *iSC.MEB* clusters on WT and CTL OCT samples 13
S11 MA plots of male vs female differential expression results for each annotated cluster . . . 14
S12 Barcode plots of sex-specific signature in male vs female differential expression results for each annotated cluster 15
S13 Spatial clustering of *T-bet* KO and CTL OCT samples 16
S14 Dot plot of Gene Ontology enrichment analysis results for *T-bet* KO vs CTL comparison in T cell cluster 17
S15 Permeabilisation time course results obtained from the Visium Spatial Gene Expression Tissue Optimization kit 18

Refer to Additional file 1: Table S1.

Table S1. Summary of experiments, separated by poly-A-based experiments (green) and probe-based experiments (blue). A separate single-cell experiment is included. Within OCT/FFPE blocks, experiments are ordered by date of sequencing run (Seq. date). Mouse sample IDs are grouped by genotype, where 'KO' is knockout of gene *T-bet*, CTL is control, and WT is wildtype. The OCT manual experiment was sequenced over 2 runs, on 5-Dec-22 and 3-Jan-23, to get the desired number of reads for each sample, except for samples 545 and 708 (marked by asterisks), which were only sequenced once on 5-Dec-22. The earlier date is used in the table. The CytAssist (CA) experiment includes 4 samples in the same run; they are presented as separate rows for OCT CA and FFPE CA.

Refer to Additional file 1: Table S2.

Table S2. Summary of RNA quality, sequencing saturation, bleeding rate, UMI counts and spots, separated by poly-A-based experiments (green) and probe-based experiments (blue). RIN values for OCT samples and DV200 scores for FFPE samples are provided. RNA bleeding rates were calculated using *SpotClean* version 1.6.1. Numbers (n) and proportions (p) of UMI counts per spot, valid UMI counts under tissue, and spots under tissue, before and after quality control, are reported. The CytAssist (CA) experiment includes 4 samples in the same run; they are presented as separate rows for OCT CA and FFPE CA.

Refer to Additional file 1: Table S3.

Table S3. A list of marker genes for cell types or different tissue regions expected in the mouse spleen selected from previous studies and existing literature. These marker genes were used for cluster scoring of spatial clusters obtained from *iSC.MEB* to inform their annotation.

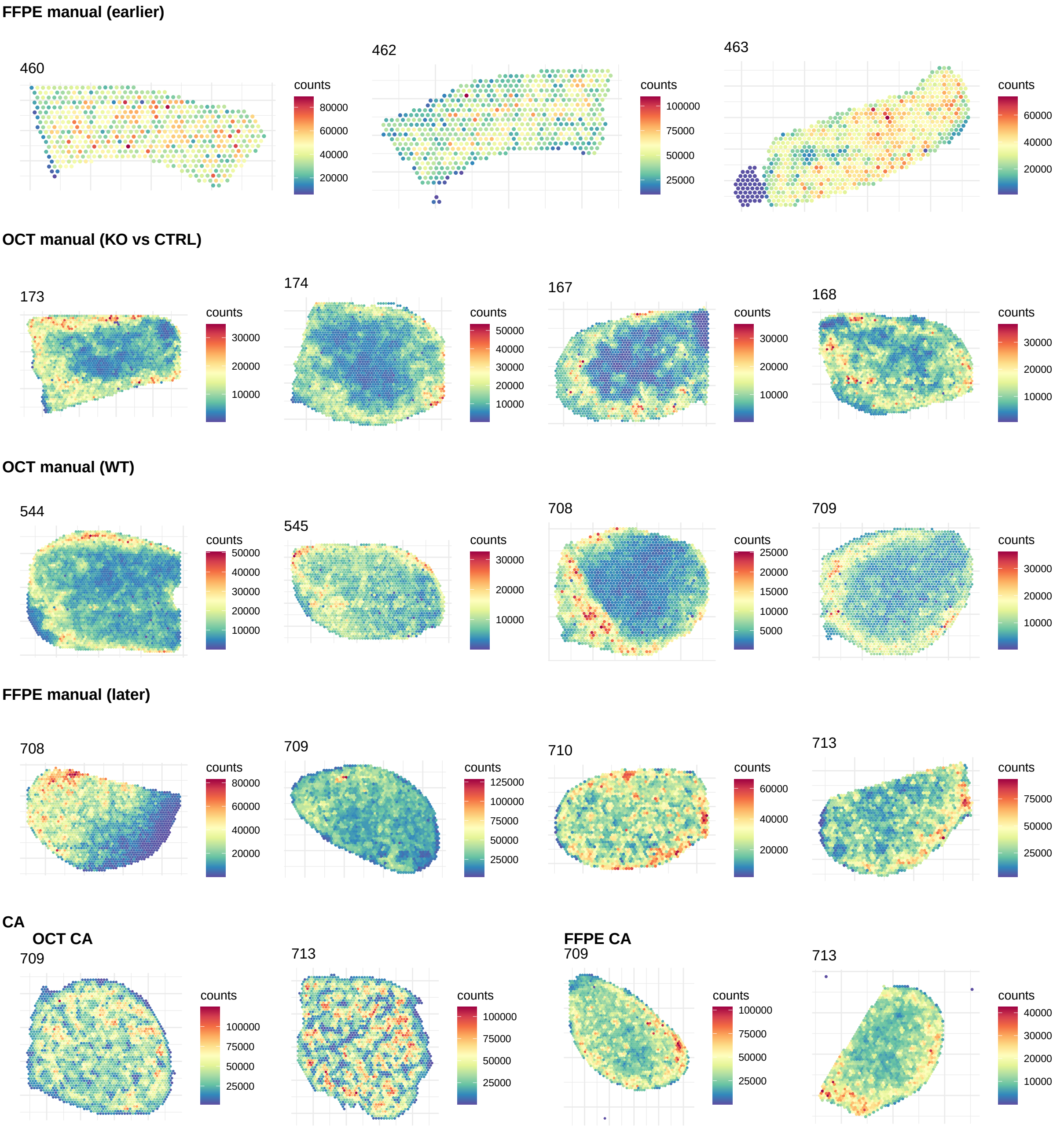

Fig. S1. Plots showing the spatial distribution of UMI counts for all samples in the study, grouped by sample type.

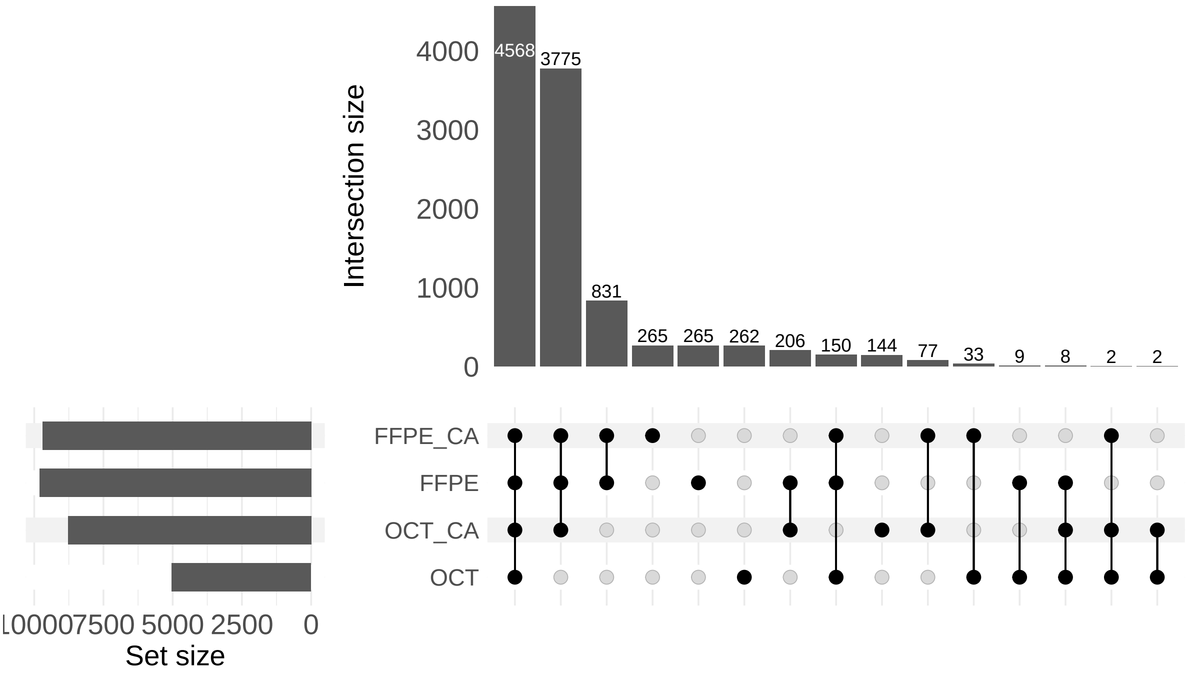

Fig. S2. Upset plot showing the overlap of detected genes in all WT samples, categorised by sample type. Detected genes are defined as genes with a count of 3 or more in at least 1% of spots.

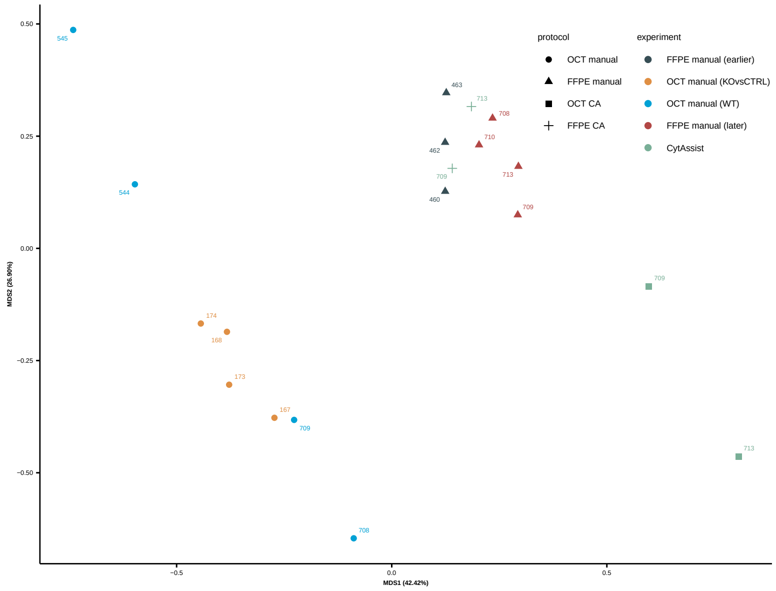

Fig. S3. MDS plot of pseudo-bulk counts for all samples based on the 488 SVG genes detected across all wild-type samples (as shown in Figure 3c).

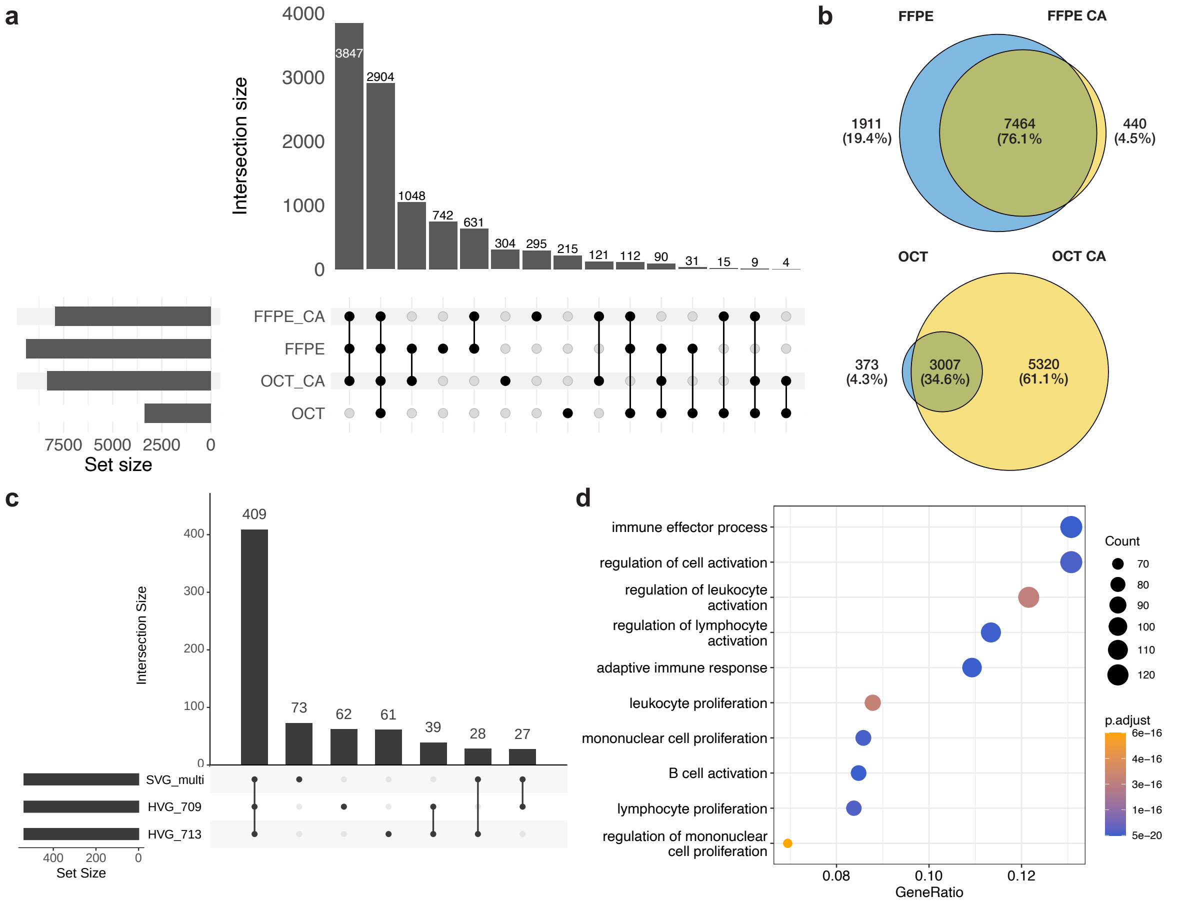

Fig. S4. Analysis of SVGs across all sample types and HVGs in FFPE CA samples. (**a**) Upset plot of the overlap of all SVGs in each sample type for WT samples only. (**b**) Venn diagrams showing unique and overlapping SVGs between FFPE samples and between OCT samples, with and without CytAssist, among all SVGs. (**c**) Upset plot showing how variable genes intersect in FFPE CA samples, at the point of highest overlap between all gene lists (537 genes). (**d**) Heatmap showing the most highly enriched Gene Ontology (GO) terms of the top 1,000 SVGs in FFPE CA samples. 'GeneRatio' denotes the ratio of top SVGs to the total number of SVGs for a particular GO term. An adjusted p-value cutoff of 0.05 was used.

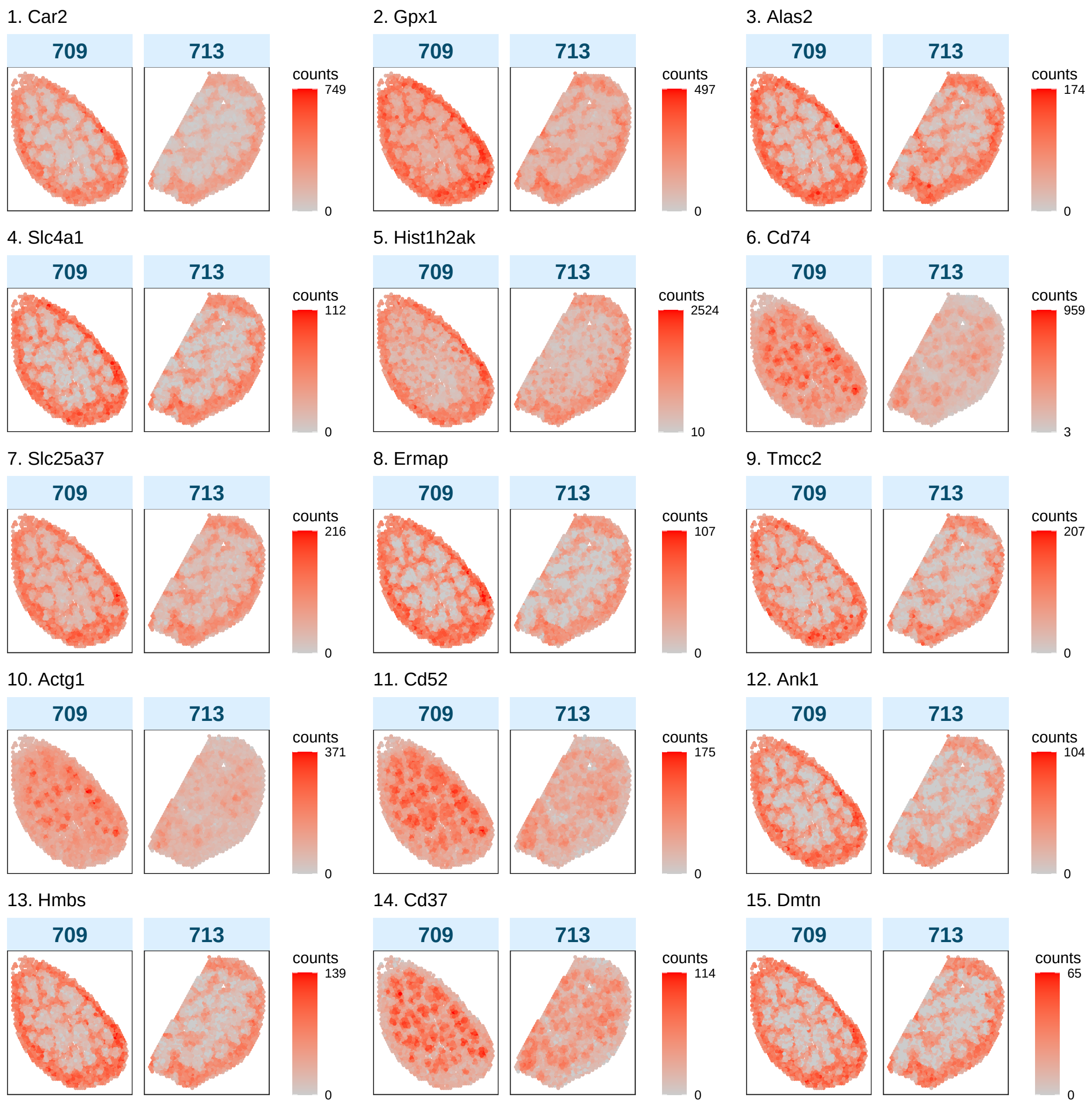

Fig. S5. Plots displaying the spatial expression of the top 15 SVGs in FFPE CA samples, identified by *nnSVG*. Heatmaps display UMI counts per spot.

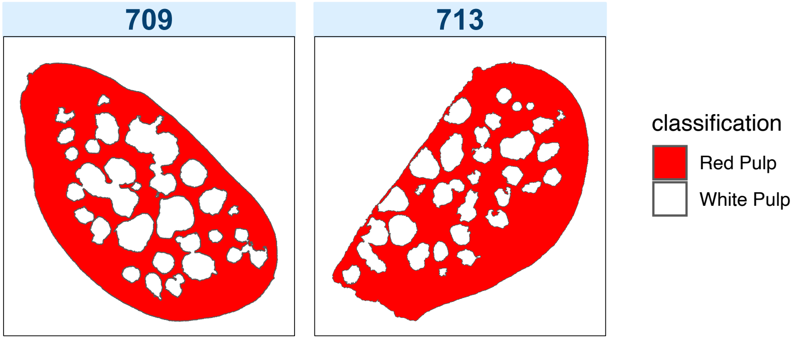

Fig. S6. Plots showing predicted red pulp and white pulp regions in FFPE CA samples. Annotations were generated by a classifier trained with a series of pathology images imported into *QuPath* version 0.4.3.

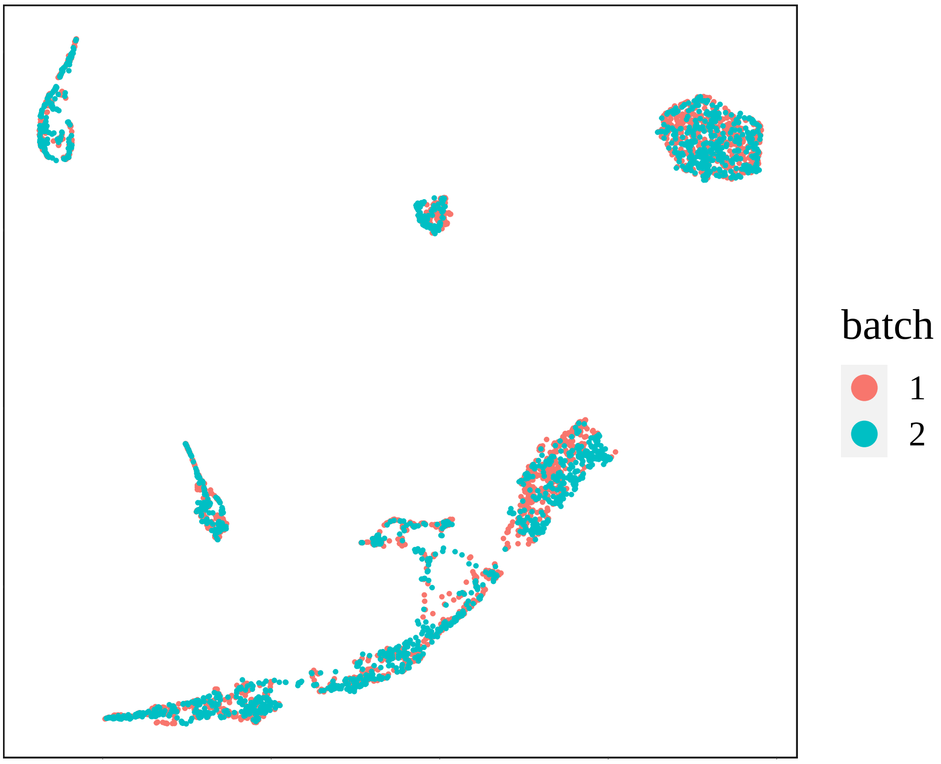

Fig. S7. A UMAP plot generated with the *iSC.MEB* package showing the spatial distribution of FFPE CA sample batches, with 'batch' referring to the samples where 1 is sample 709 and 2 is sample 713.

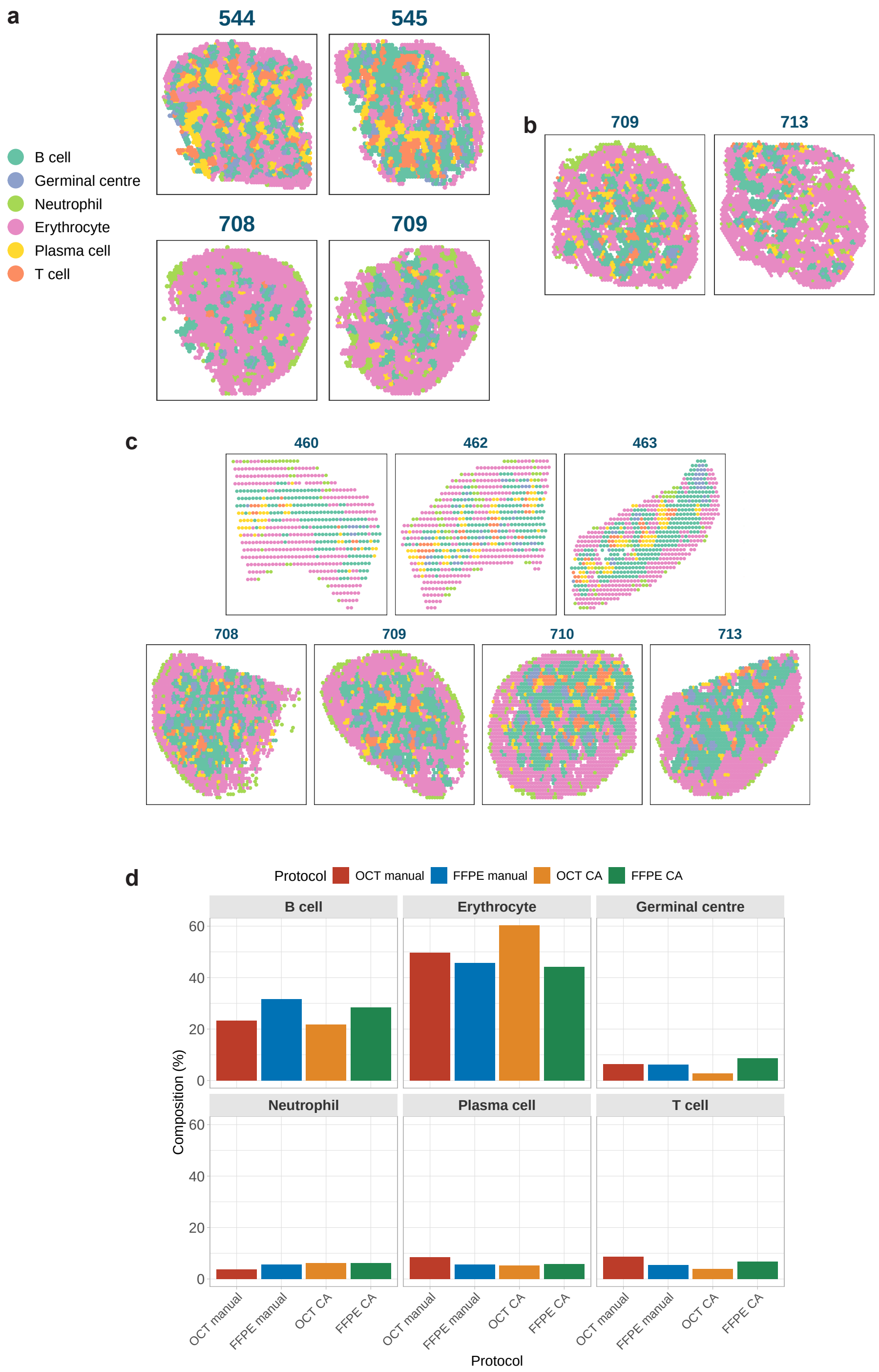

Fig. S8. Cell type annotation and composition across all protocols. (**a**) OCT manual samples with each spot annotated following deconvolution and marker gene expression analysis, using spatial clusters. (**b**) OCT CA samples with each spot annotated following deconvolution and marker gene expression analysis, using spatial clusters. (**c**) FFPE manual samples with each spot annotated following deconvolution and marker gene expression analysis, using spatial clusters. (**d**) The cell type composition of spatial clusters in each protocol, shown as percentages of the total spot count across all samples in the protocol.

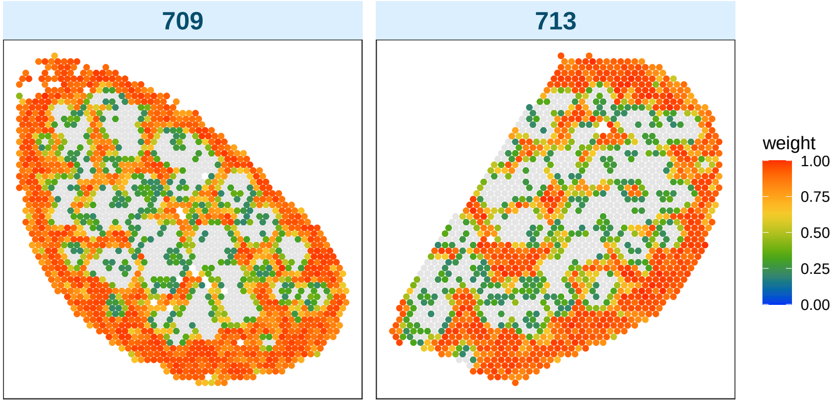

Fig. S9. Erythrocyte classification following deconvolution of FFPE CA samples. Plots show the spatial distribution of normalised confident weights, predicting erythrocyte proportions, in each spot. Weights were generated by *spacexr* version 2.2.1 during cell type deconvolution of FFPE CA samples.

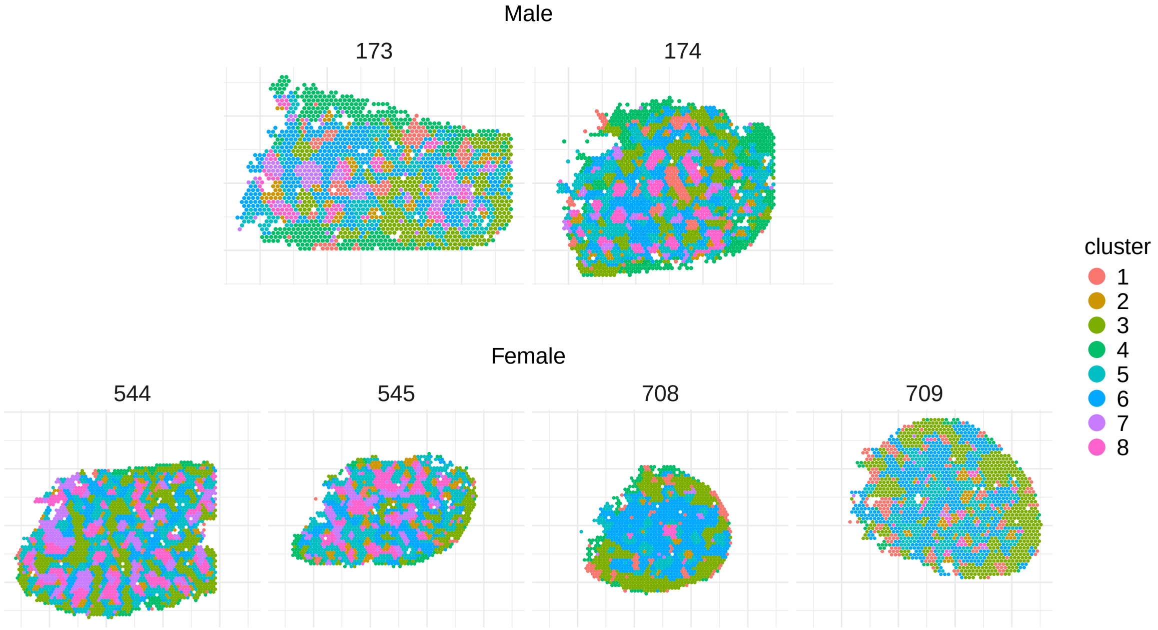

Fig. S10. Spatial plot of *iSC.MEB* clustering result for each WT and CTL OCT spleen sample. Male samples are shown in the top row, and female samples in the bottom row.

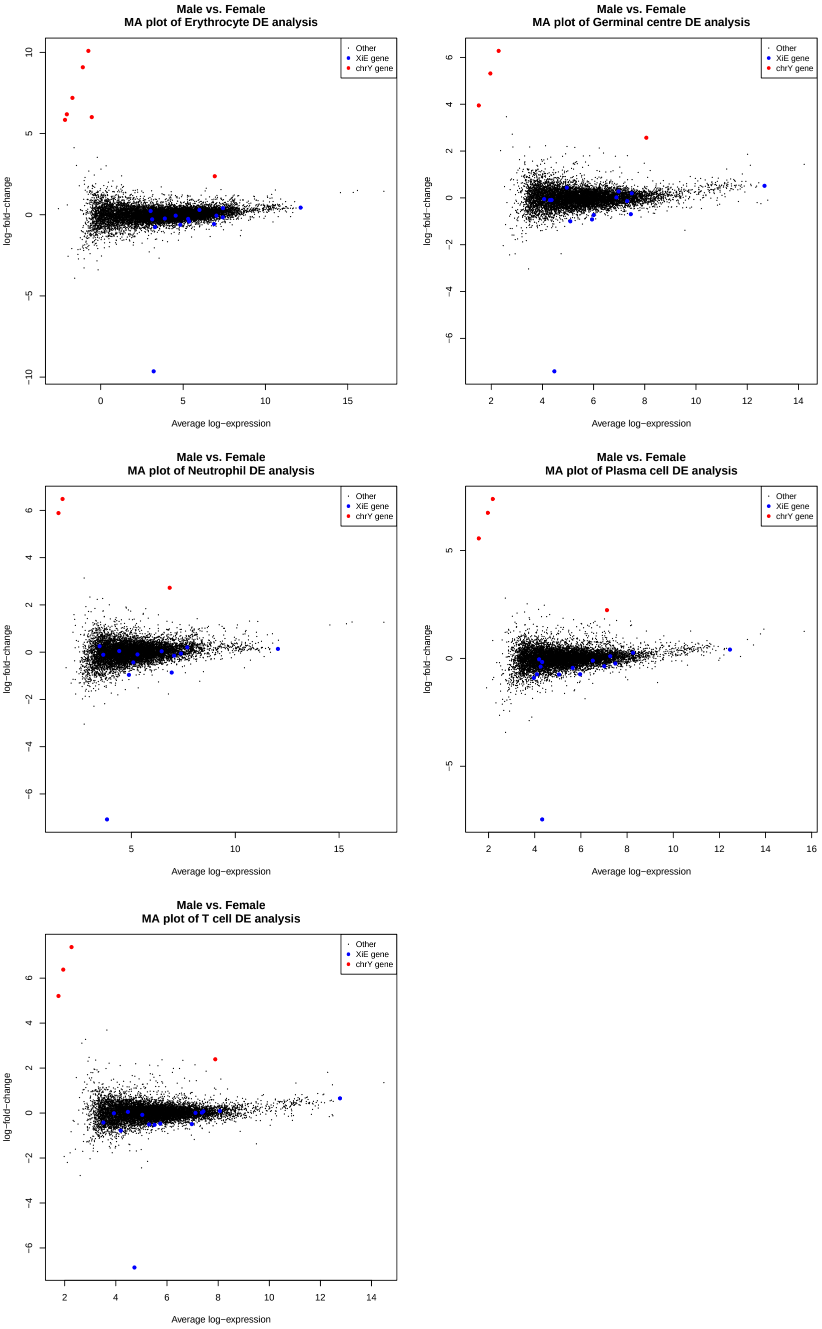

Fig. S11. Log-fold-change vs mean expression (MA) plots of the differential expression analysis between male and female samples based on pseudo-bulk counts for different clusters. Sex-specific genes are highlighted in colour (blue: genes that escape X inactivation in mouse spleen, red: chromosome Y genes).

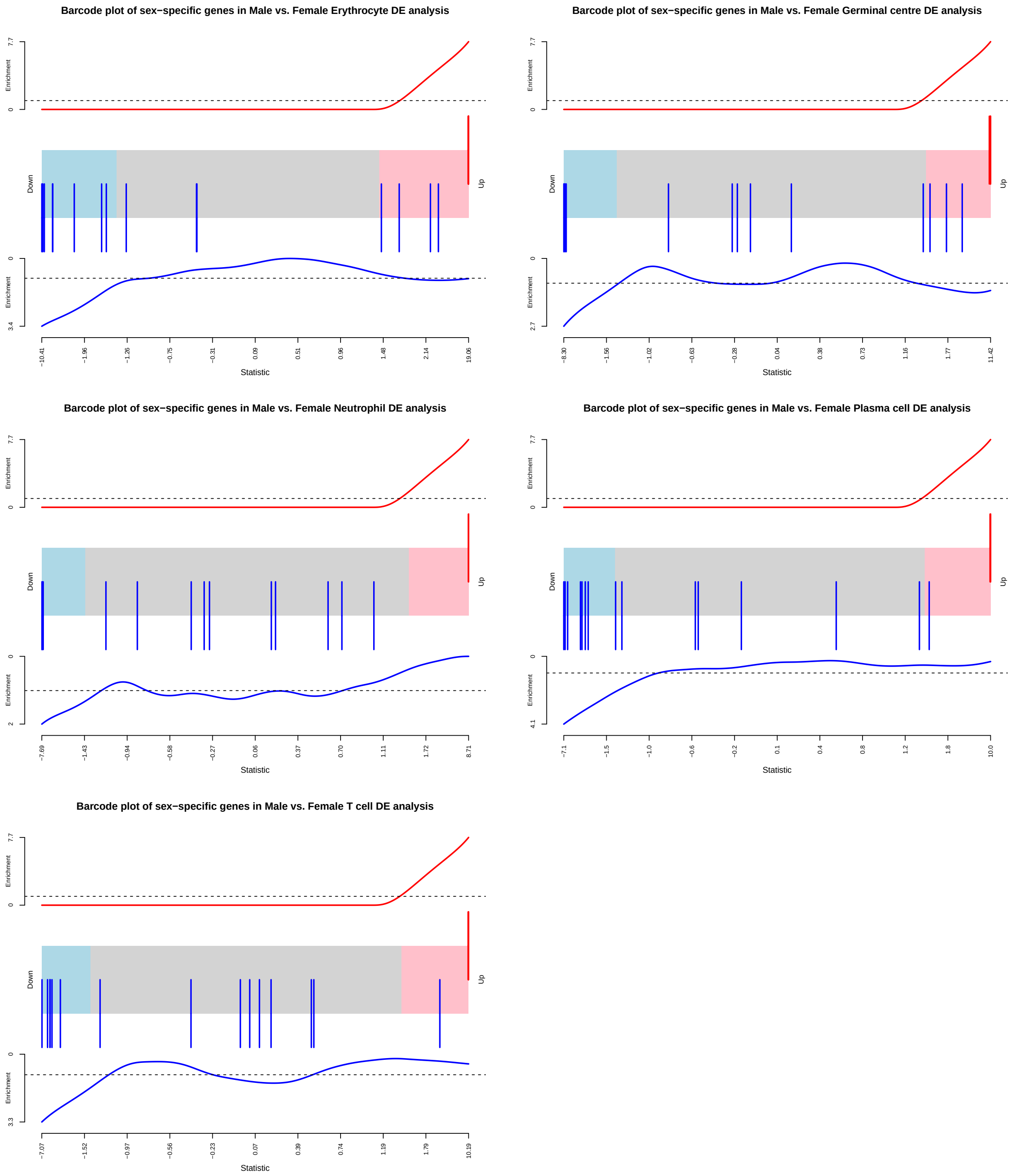

Fig. S12. Barcode plot of male vs female differential expression analysis results from pseudo-bulk counts for each cluster, with the ranks of sex-specific signature genes highlighted in colour (blue: genes that escape X inactivation in mouse spleen, red: chromosome Y genes). *ROAST* enrichment p-values for this signature for each cluster are available in Table 1.

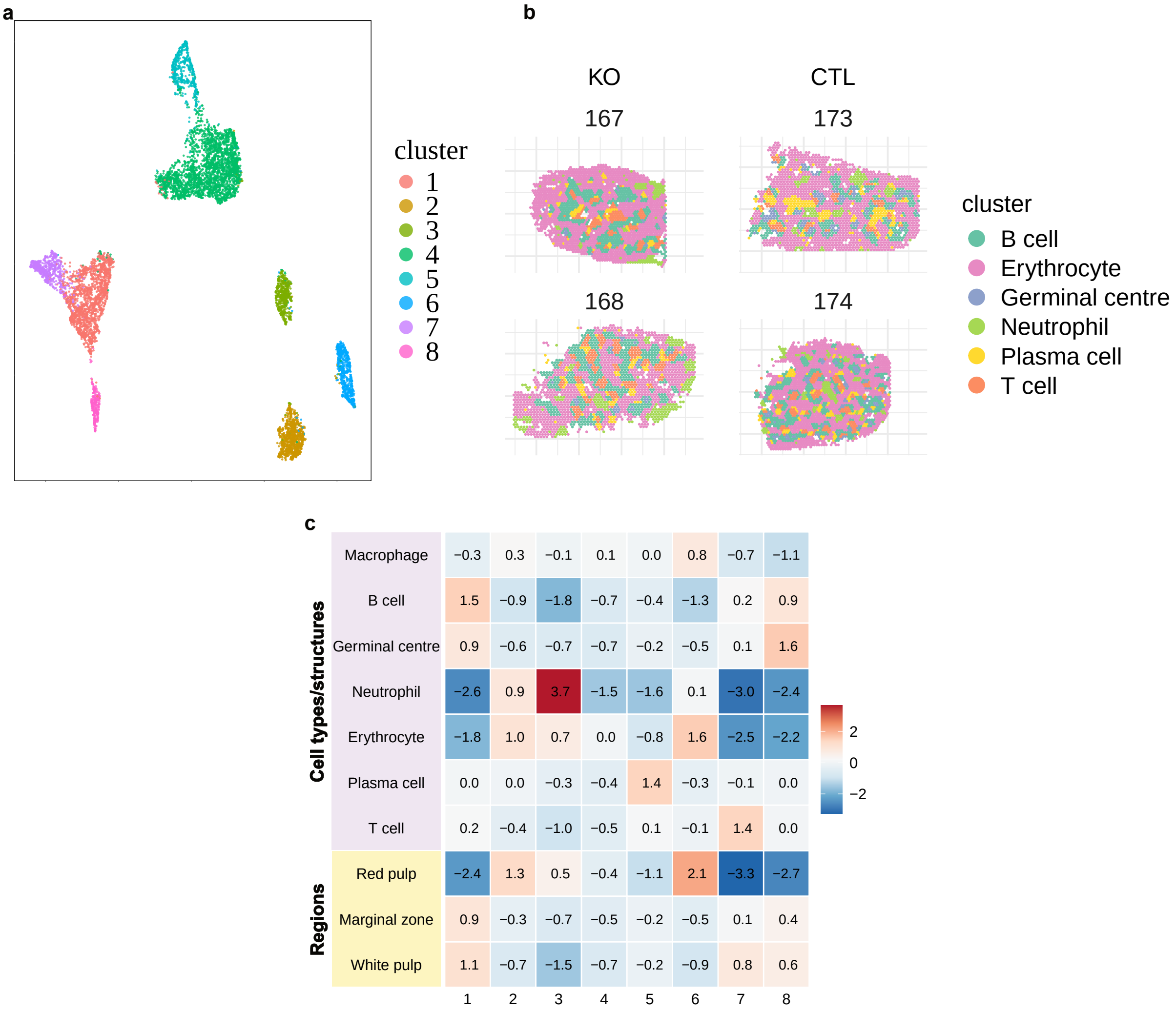

Fig. S13. (**a**) UMAP of *iSC.MEB* clustering result for the KO and CTL OCT samples. (**b**) Spatial plot of *iSC.MEB* clustering result for KO and CTL samples, with KO samples displayed on the left and CTL samples on the right. (**c**) Heatmap of expression scores generated using marker genes for different cell types or tissue regions expected in the spleen for each spatial cluster compared to all other clusters.

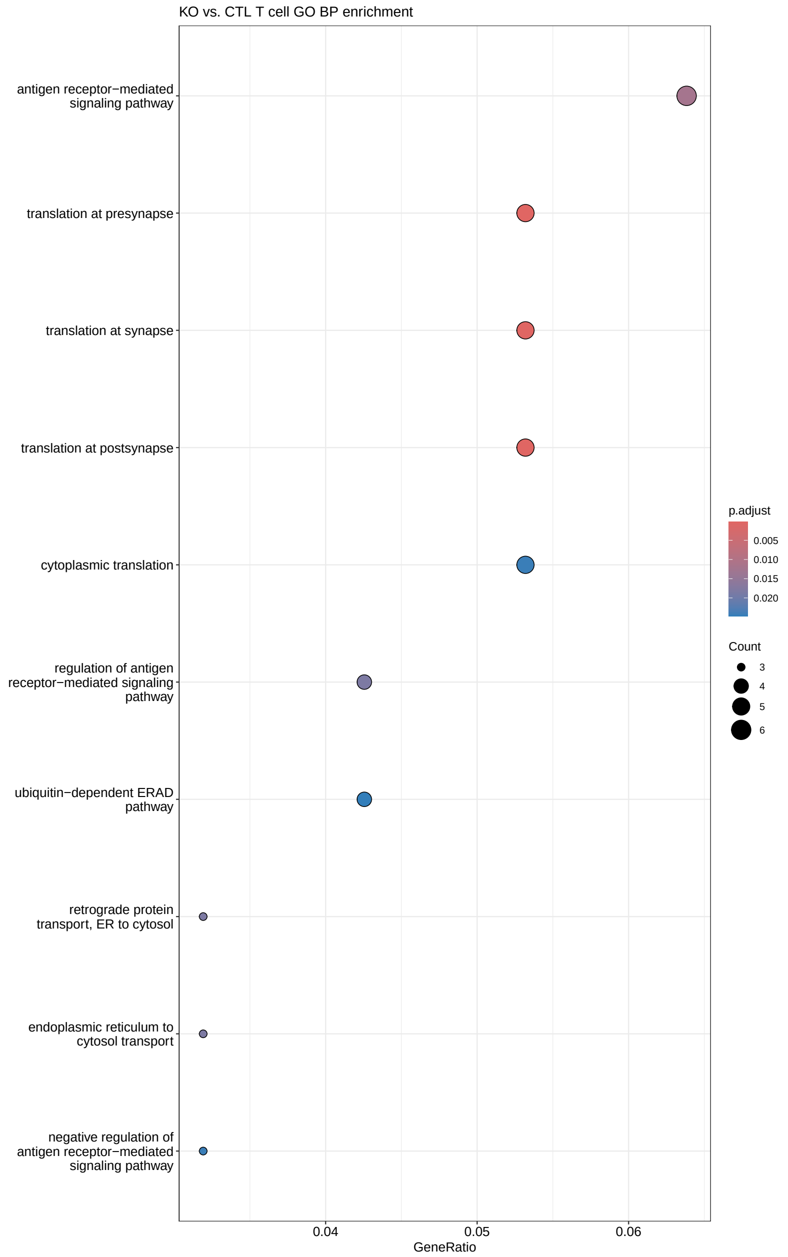

Fig. S14. Dot plot showing the top-ranked Gene Ontology (GO) biological process terms enriched in the T cell cluster for the KO vs CTL comparison. The top 10 most significant terms are plotted, coloured by the adjusted p-value and sized by the number of differentially expressed genes for the term. The x-axis represents the ratio of genes in the term over the total number of differentially expressed genes.

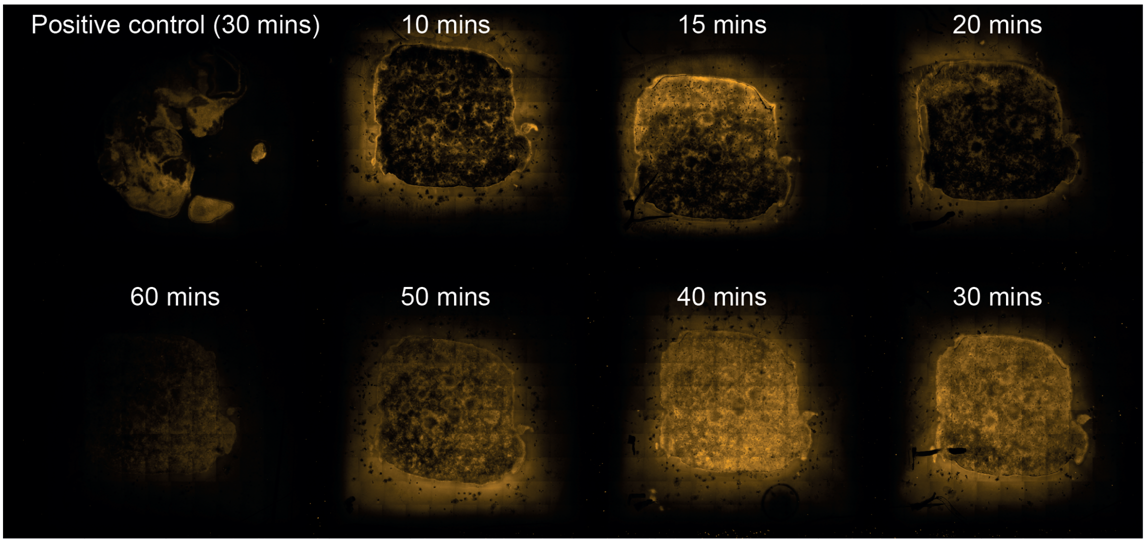

Fig. S15. Permeabilisation time course results obtained from the Visium Spatial Gene Expression Tissue Optimization kit. Mouse E12.5 embryo was used as a positive control, with a permeabilisation time of 30 mins. Spleen tissue sections are labelled with increasing permeabilisation times, from 10 mins up to 60 mins.
