# Supplementary material for: Benchmarking spatial transcriptomics technologies with the multi-sample SpatialBenchVisium dataset
Source: Genome Biol. 2025 Mar 28;26:77. doi: 10.1186/s13059-025-03543-4 (PMC11954323; doi:10.1186/s13059-025-03543-4)
Supplement: Supplementary file 3 — Additional file 3: Review history. [file 13059_2025_3543_MOESM3_ESM.docx]

Review History

**First round of review**

**Reviewer 1**

**Were you able to assess all statistics in the manuscript, including the appropriateness of statistical tests used?**

Yes, and I have assessed the statistics in my report.

**Comments to author:**

Comments to Author
Comments to Author General comments: The manuscript ‘Spotlight on 10x Visium: a multi-sample protocol comparison of spatial technologies’ by Du et al. introduces a multi-sample dataset profiling spatial gene expression of mouse spleen tissue with various combinations of sample handling and protocol combinations with the 10x Genomics Visium platform. The authors compare metrics for quality control along with a comparison of downstream analyses tasks with existing computational methods. Overall, I thought the authors did a thoughtful and thorough analysis of QC metrics between the platforms, but I was less convinced of the comparisons of the downstream analysis. However, I don’t think the comparisons of downstream analyses were the major point of their important contributions here (and this did not diminish my enthusiasm for the paper). My comments below mostly are focused on asking for additional clarification or pushing the authors to do a bit more data exploration that I think would be helpful to the community. In summary, the authors have produced a useful dataset that will be helpful to the community as we as researchers try to keep up with the fast-moving landscape of technological developments. I applaud them for their contributions!

Major:

- One plot I would appreciate the authors making to help assess the variation between the differences in sample handling and protocol combinations is the following. Could the authors create pseudobulk profiles for each cell type / spatial domain and capture area? 13 samples processed in 4 ways then should be a total of 52 * number of spatial domains pseudobulk profiles. The reason for this is I would appreciate getting a sense of how similar / dissimilar these 4 protocols are and this is a great plot to assess this variation

- The authors mentioned the spots beyond the tissue boundary were filtered out. However, I think that is an important analysis to further explore here. It has been noted in the literature that using the OCT protocol that RNA bleeds beyond the tissue boundary leading “spot swapping” (PMID: 35624112). Could the authors assess whether this is corrected (or similar) using one of the other protocols (FFPE, OCT CA, FFPE CA)? This would be very helpful for the field (for those both developing the spatial technologies, but also those analyzing the data). Ion full transparency, this might have been captured by the section titled “CytAssist facilities the capture of more reads under tissue”, but I could only find one plot (Figure 1e) referring to this. If so, please clarify. I would appreciate more exploration here to understand this important issue.

- The authors make a compelling case for using probe-based protocols as they have higher numbers of UMI and higher mapping confidence. But could the authors talk about (maybe in the discussion?) the disadvantages of probe-based protocols? They did mention the lack of MT genes in the probe-based protocols. That seems to be an important difference.

- Figure 3 was hard to follow when the clusters were not interpreted to meaningful cell type names. I see this was done for Figure 3h, but I think help understanding what the clusters are in the above figures would be helpful. Or at least some marker genes for the clusters in the UMAP?

Minor:

- The authors comment that “by far the most popular sequencing-based method at present is the commercially available Visium method”. While I agree with you, I haven’t seen formal data on it. Would it be possible to quantify in that some way?

- The authors name their reference dataset SpatialBench, but I think it’s important to emphasize that this dataset is was generated from 1 company (10x Genomics) on versions of their Visium platform. That does not come across in the name of the dataset and might be useful to consider incorporating (e.g. SpatialBenchVisium). It also leaves open the door for a similar comparison based on other platforms in the future.

- This is likely out of scope, but have the authors considered generating long-read RNA-seq data from the cDNA libraries of these tissue profiled?

- The legend labels (descrbing the differences in colors) in Figure 1c and d are not clear. For example, I couldn’t remember what the difference was between FFPE (1) and FFPE (3). Similar comment for the “KOvsCTRL (2)” vs “OCT (2)”, etc. Could this be made more clear? I also struggled with trying to understand Figure 1e for the same reasons. The shapes were clear, but I was flipping back and forth trying to understand the colors. Like the higher y-axis for the black is interesting, but that I’m assuming is mostly an outlier and should be closer to the red points as they are both FFPE?

- The authors switch between using “Probe-based” and “CytAssist” in the section headers in the results sections. Could these be made uniform one way or the other?

- The venn diagrams in Figure 2e and 2f are misleading as visually they show a similar story, but numbers wise they are very different. Same for Figure 3d.

**Reviewer 2**

**Were you able to assess all statistics in the manuscript, including the appropriateness of statistical tests used?**

Yes, and I have assessed the statistics in my report.

**Comments to author:**

Du et al. present SpatialBench, a multi-sample, multi-protocol 10X Genomics Visium dataset that can be used as a practical guide to design and analyze spatial transcriptomics experiments.

The manuscript is well-written and presents a comprehensive dataset that convincingly compares different versions of the 10X Genomics Visium method applied to malaria-infected mouse spleen samples.

The first part of the manuscript is very useful as it systematically compares CytAssist versus manual Visium as well as FFPE versus fresh frozen samples. These results can serve as a guideline for researchers needing to decide on which protocol to use for their experiments.

In the second part of the manuscript, the authors showcase an R/Bioconductor-based workflow representing a typical downstream analysis of multi-sample, multi-condition Visium data: they first show that their approach can distinguish sex-specific gene expression in control samples and then apply their pipeline to comparing knock-out versus control infected samples, showing a good concordance with a previously published bulk RNA-seq experiment.

Overall, I enjoyed the paper and I believe that the SpatialBench dataset has the potential to be a valuable resource for the research community. However, to live up to this expectation, the authors should address the following points.

1. To maximize the usability of the dataset for methods' benchmarking, I would strongly encourage the authors to package the data in a user-friendly format, such as an R or Bioconductor data package, as previously done by others with, e.g., the SpatialLIBD data (https://bioconductor.org/packages/release/data/experiment/html/spatialLIBD.html). Ideally, interested researchers could load the data in standard format (e.g., SpatialExperiment or similar) with one line of code, as done in SpatialLIBD.

2. In the same spirit, I noticed that the data on GEO only include the "hires" and "lowres" versions of the H&E image from Visium. I suspect that the authors used a much higher resolution of the image when performing their QuPath analysis (sometimes referred to as "original resolution"). Was that the case or was the "hires" image used? If the former, it would be important for the reproducibility of the paper and for the importance of the resource for the authors to share (e.g., through FigShare or Zenodo) the original H&E images. This will be important for researchers wanting to benchmark methods that make use of imaging features in addition to gene expression for the analysis of Visium data.

3. It would be useful to have a better global picture of the relative contribution of technical and biological variables to the overall variation observed in the data. For instance, are FFPE samples from different genotypes more or less similar to control samples assayed with FFPE or OCT techniques? One way of achieving this could be to look at the variance explained by each variable (e.g., sex, genotype, FFPE/OCT, CA/manual, …). This can be done at the single-cell level, e.g., by looking at the variance explained by each principal component of the merged data and relating that to the above-mentioned variables, or perhaps more easily, at the pseudo-bulk level, by looking at multi-dimensional scaling plots color-coded by the different variables. An overall assessment of batch effects and what aspects of the protocols have a larger effect on separating batches would also be useful.

4. The authors did a great job at comparing the different protocols in terms of sample quality (using per-spot QC metrics), but it would be useful to look at the impact of the protocol in downstream results, e.g., clustering. One way to achieve this would be to look at cluster concordance across protocols and cluster stability within each protocol. For instance, the authors could perform cluster analysis for each protocol separately and then look at how the cell type distributions change between them; this analysis will likely be confounded with the biological differences between genotypes and the individual morphology of the samples, but it could give a rough idea of whether any strong bias is present in any of the methods. Alternatively, they could look at the concordance between marker gene lists identified downstream of clustering performed separately for each protocol.

5. The authors should improve Figure 1b to give a clearer overview of the experimental design. It took me a lot of back and forth between the main text, the methods section, and the supplementary tables to clearly understand how the experiment was designed. In my opinion the "Sample preparation, library construction, sequencing, data analysis" part of Figure 1b is unnecessary and remove it would make room for a more detailed version of the "Experimental design" table, perhaps merging information from Figure 1b and Supplementary Table S1. It would also be useful to clarify upfront what "KO" and "CTL" mean in terms of their genotype.

Minor issues:

- I found it confusing to refer to the individual experiments as "Experiment 1", "Experiment 2", etc. Could the authors use more descriptive names? Such as "FFPE manual", "FFPE CA", etc?

**Reviewer 3**

**Were you able to assess all statistics in the manuscript, including the appropriateness of statistical tests used?**

Yes, and I have assessed the statistics in my report.

**Comments to author:**

This benchmarking study by Du, Wang, Law, Ammann-Zalcenstein et al. has potential to aid researchers in selecting the appropriate 10x Visium-based approach for Spatial Transcriptomics. The authors make a rather extensive comparison of four approaches used on the same tissue or same tissue type. The experimental workflow is easy to follow, and the results are well-described in both text and figures. Unfortunately, I am skeptical about the comparison due to some major concerns stated below.
The presented data analysis workflow and results do not pose any significant advancements, which in my opinion is acceptable if the aim of the manuscript is to benchmark the wet lab protocols. However, unless the benchmarking part can be heavily revised, I suggest that the authors change the focus of the study and make it more biology-oriented. Surely, there are findings to be mined from this rich dataset.

I have used the following abbreviations in my comments: page X, column Y, row Z -> pXcYrZ. Other abbreviations are equivalent to those used in the manuscripts.

Major comments:

1) Comparing UMIs.
UMIs per spot is used as sensitivity (and performance) measure throughout the manuscript. However, there may be an important difference in what a UMI represents in 10x's poly-A versus probe-based approaches. In the former, a UMI always equals one captured transcript whereas in probe-based protocols there are up to three probe pairs per target transcript, all with different UMIs. Resultingly, the same number of original transcripts profiled with the two approaches may lead to three times more UMIs in the FFPE and CA experiments compared to FF (without CA). The number of probes (and UMIs) per target are different for the different versions and the issue may in any case be resolved bioinformatically. The fact that this is not mentioned (nor what probe-set was used) makes me doubt the entire comparison.

2) Tissue freezing and RNA quality.
The freezing protocol can affect RNA quality, and I find it unfortunate that the authors do not give any details on the freezing strategy. Also, I cannot find any measurement of the RNA quality in the fresh-frozen samples, which can strongly impact the performance of the poly-A capture protocol. RIN values should be provided for the FF samples and DV50 for FFPE.

3) Sub-optimal permeabilization.
The authors acknowledge that permeabilization in the FF experiments may have been suboptimal. Tissue optimization is undoubtedly a challenge when working with the FF material, but for a benchmarking study I am concerned that a suboptimal permeabilization will skew the whole comparison.

4) Sequencing depth.
"Samples were sequenced according to 10x's guidelines". This is indeed a good starting point, but samples differ in complexity and RNA quality. To really assess the performance of the tissue handling protocols, samples should be sequenced to saturation. At the very least, an estimation of sequencing saturation per sample should be provided.

5) Adaptive immune receptor variable genes.
I assume IGHV..., IGLV…, TRBV… etc. were among the genes that were exclusively detected in OCT (poly-A-based) samples, as these are not included in the probe set. The authors should comment on whether these genes were detected, and make sure to exclude them prior to clustering as the can drive cluster formation in a way that is not biologically meaningful. Depending on their abundance in the data, there is however the potential to use them for VDJ analysis, which should also be commented on.


Minor comments:
6) Definition of detected genes.
There is a significant difference in how many genes were detected depending on cut-off ( ≥3 UMIs in at least 10% or 1% of spots under tissue). How was the cut-off selected?

7) Detected genes in OCT samples.
On p4c2r2 the authors state that across the samples, 1,636 genes were detected in OCT samples, with a reference to Fig2d. This panel however shows only WT samples. From Fig2d, I end up with 1,720 genes when summarizing, same as in Fig2f. Please clarify, are 1,636 the mean number across all samples? Also, please clarify what samples are shown in Fig2e-f (I'm guessing wt samples, same as in Fig2d?).

8) Overlap of genes in probe-based samples.
"Similar numbers in the overlap of probe-based samples were observed for both thresholds, 4,000 genes." (p3c2r29) From Fig2d and figS2 my understanding is that the overlap is 5,142 and 8,343 respectively. What is the number 4,000 derived from?

9) "We have created SpatialBench, a unique multi-sample spatial benchmarking dataset while profiles the mouse spleen as a reference tissue, generated using 10x Genomics' Visium spatial technology." (p10c1r23-26). I do not understand this phrase.

10) Typo: P4c2r58 "along" -> "alone"

11) P8c1r61: duplication of "the"

12) "In these samples, we also detected a bias showing higher UMI counts along tissue edges." (p10c1r54-54) "These samples" refer to OCT (poly-A based) samples, yes? Please clarify to avoid confusion.

13) Fig 3g seems to be referenced prior to Fig 3f in the text. It would be easier to follow if figures followed the same order as r

**Authors Response**

**Point-by-point responses to the reviewers’ comments:**

**Reviewer #1:**

The manuscript ‘Spotlight on 10x Visium: a multi-sample protocol comparison of spatial technologies’ by Du et al. introduces a multi-sample dataset profiling spatial gene expression of mouse spleen tissue with various combinations of sample handling and protocol combinations with the 10x Genomics Visium platform. The authors compare metrics for quality control along with a comparison of downstream analyses tasks with existing computational methods. Overall, I thought the authors did a thoughtful and thorough analysis of QC metrics between the platforms, but I was less convinced of the comparisons of the downstream analysis. However, I don’t think the comparisons of downstream analyses were the major point of their important contributions here (and this did not diminish my enthusiasm for the paper). My comments below mostly are focused on asking for additional clarification or pushing the authors to do a bit more data exploration that I think would be helpful to the community. In summary, the authors have produced a useful dataset that will be helpful to the community as we as researchers try to keep up with the fast-moving landscape of technological developments. I applaud them for their contributions!

**Response:** We thank the reviewer for their supportive feedback on our work and constructive comments.

Major:

- One plot I would appreciate the authors making to help assess the variation between the differences in sample handling and protocol combinations is the following. Could the authors create pseudobulk profiles for each cell type / spatial domain and capture area? 13 samples processed in 4 ways then should be a total of 52 * number of spatial domains pseudobulk profiles. The reason for this is I would appreciate getting a sense of how similar / dissimilar these 4 protocols are and this is a great plot to assess this variation.

**Response:** Thank you for the suggestion. We have added a pseudo-bulk MDS plot which shows the relationship between samples prepared and processed in different ways. This has been included as the new Supplementary Figure S3 and is also shown below. Briefly, this figure shows separation based on library preparation method (poly-A versus probe-based) explaining the most variation in dimension 1 and 2.

−0.5	0.0	0.5

**MDS2 (26.90%)**

- 0.25
- 0.50

0.50

0.25

0.00

544

709

174

168

167

709

708

experiment

FFPE manual (earlier) OCT manual (KOvsCTRL) OCT manual (WT) FFPE manual (later) CytAssist

545

protocol

OCT manual FFPE manual OCT CA FFPE CA

463

713

708

710

462

709

713

173

709

713

460

**MDS1 (42.42%)**

**Supplementary Figure S3.** MDS plot of pseudo-bulk counts for all samples based on the 488 SVG genes detected across all wild-type samples (as shown in Figure 3c).

- The authors mentioned the spots beyond the tissue boundary were filtered out. However, I think that is an important analysis to further explore here. It has been noted in the literature that using the OCT protocol that RNA bleeds beyond the tissue boundary leading “spot swapping” (PMID: 35624112). Could the authors assess whether this is corrected (or similar) using one of the other protocols (FFPE, OCT CA, FFPE CA)? This would be very helpful for the field (for those both developing the spatial technologies, but also those analyzing the data). Ion full transparency, this might have been captured by the section titled “CytAssist facilities the capture of more reads under tissue”, but I could only find one plot (Figure 1e) referring to this. If so, please clarify. I would appreciate more exploration here to understand this important issue.

**Response:** We thank the reviewer for their question. After using SpotClean to estimate contamination levels, we see that the use of CytAssist tissue placement platform has significantly reduced the spot swapping or RNA bleeding effect. The average bleeding rates are 0.11 for CA, 0.52 for FFPE manual, and 0.47 for OCT manual. We also added the bleeding rates to Supplementary Table S2 and refer to SpotClean in the Methods.

The relevant text, with major edits highlighted in red:

1. We also assessed the extent of spot swapping with SpotClean, an effect where RNA bleeds to surrounding spots, leading to a spot containing UMI's from different origins which can confound downstream analyses. This effect has been seen with the OCT manual protocol and our results suggest that the use of CytAssist tissue placement

may correct for it. The average bleeding rate for our OCT manual samples was 0.47 and for the FFPE manual samples it was 0.52. For CA samples, this was much lower at 0.11 (Supplementary Table S2).

2. SpotClean version 1.6.1 was used to calculate the RNA bleeding rate for each sample using the main function. Individual samples were pre-processed following a standard Bioconductor workflow for spatial transcriptomics analysis.

- The authors make a compelling case for using probe-based protocols as they have higher numbers of UMI and higher mapping confidence. But could the authors talk about (maybe in the discussion?) the disadvantages of probe-based protocols? They did mention the lack of MT genes in the probe-based protocols. That seems to be an important difference.

**Response:** We thank the reviewer for raising this important point. In the discussion, we also noted the absence of several immune-related and certain cell type marker genes from the probe set, which may have implications for the biological aspects of our study. We have added further comments on the lack of genetic variation in probe-based data.

The relevant text, with major edits highlighted in red:

It is worth noting that, however, a number of genes including some in the probe set were only detected in OCT samples, after filtering. Of these genes, there were several related to immune response and red pulp. Moreover, certain cell type marker genes were absent from the probe set. Without significant efforts to design custom probes, probe-based data also lack the ability to measure genetic variation in transcripts or isoform-level expression variation if long-read sequencing were applied to the library, and are only available for human and mouse species at present, which will affect the derivation of meaningful biological insights in many applications. These considerations will motivate the use of poly-A capture in some studies despite its poorer sensitivity.

- Figure 3 was hard to follow when the clusters were not interpreted to meaningful cell type names. I see this was done for Figure 3h, but I think help understanding what the clusters are in the above figures would be helpful. Or at least some marker genes for the clusters in the UMAP?

**Response:** We apologise for the confusion. The cluster numbering in Figures 3e and 3f are consistently numbered in both the UMAP and the heatmap. The finer grade cell type ! small-scale structure labels versus the broader tissue region annotations were derived from the marker genes listed in Supplementary Table S3. The cell type ! local structure annotations are then plotted in a spatial context in Figure 3h. In the previous version of 3h we incorrectly

used the broad region label ‘red pulp’ which has been replaced with the cell type label ‘Erythrocyte’ in the updated figure. We have further distinguished the cell type and small-scale structure annotations from broad tissue regions in the spleen in Figure 3f and other relevant heatmaps (e.g. Figure 4b, Supplementary Figure S13c). The figure caption of panel 3g mentions that this result pertains to the T cell annotated cluster. We hope that this makes things clearer.

**Revised Figure 3 (e)** Top: A UMAP plot showing spatial clusters (numbered from 1 - 7) across both FFPE CA samples, identified by *iSC.MEB*. Bottom: Spatial cluster 7 projected onto tissue images. **(f)** Top: A heatmap showing scores for expression of cell type marker gene groups in each cluster (1 – 7) compared to all other clusters in FFPE CA samples. Bottom: The aggregate gene expression of the T cell marker genes in cluster 7. **(g)** A deconvolution plot of confident weights for T cells generated by *spacexr* for FFPE CA samples. **(h)** FFPE CA samples 709 and 713 with annotated spatial clusters following deconvolution and marker gene expression analysis.

Minor:

- The authors comment that “by far the most popular sequencing-based method at present is the commercially available Visium method”. While I agree with you, I haven’t seen formal data on it. Would it be possible to quantify in that some way?

**Response:** We agree and have reworded this sentence to begin with “One popular sequencing-based method” in lieu of solid data on its popularity.

- The authors name their reference dataset SpatialBench, but I think it’s important to emphasize that this dataset was generated from 1 company (10x Genomics) on versions of their Visium platform. That does not come across in the name of the dataset and might be useful to consider incorporating (e.g. SpatialBenchVisium). It also leaves open the door for a similar comparison based on other platforms in the future.

**Response:** We thank the reviewer for their feedback. We have renamed our dataset to *Spatia!BenchVisium* throughout the manuscript and modified the GitHub repository name to <https://github.com/mritchielab/SpatialBenchVisium>. This naming approach can be extended to other platforms as and when they become available (in future work).

- This is likely out of scope, but have the authors considered generating long-read RNA-seq data from the cDNA libraries of these tissue profiled?

**Response:** Yes, we have. Our first attempts at this failed though using some of the OCT poly-A libraries from this study. More effort is needed to get this working, although we note that others have succeeded (see Boileau *et a!.* 2022 Front Genet, 13: 912572. PMID: 35937994, Lebrigand et al. 2023 Nucleic Acids Res, 51(8):e47. PMID: 36928528, Engblom *et a!*. 2023 Science, 382(6675):eadf8486 PMID: 38060664).

- The legend labels (describing the differences in colors) in Figure 1c and d are not clear. For example, I couldn’t remember what the difference was between FFPE (1) and FFPE (3). Similar comment for the “KOvsCTRL (2)” vs “OCT (2)”, etc. Could this be made more clear? I also struggled with trying to understand Figure 1e for the same reasons. The shapes were clear, but I was flipping back and forth trying to understand the colors. Like the higher y-axis for the black is interesting, but that I’m assuming is mostly an outlier and should be closer to the red points as they are both FFPE?

**Response:** We apologise for the confusion. We have removed experiment numbers and now refer to experiments as their sample types (OCT manual, FFPE manual, and CA), and to individual batches more descriptively when required in the figures and the text. We have also edited Supplementary Tables S1 and S2 to use the simplified sample naming approach.

- The authors switch between using “Probe-based” and “CytAssist” in the section headers in the results sections. Could these be made uniform one way or the other?

**Response:** Our intention was to use both terms in different protocol contexts - “probe-based” when describing the samples profiled via probe-based gene selection (FFPE manual, OCT CA, and FFPE CA) and “CytAssist” when referring to tissue placement performed with CytAssist (OCT CA and FFPE CA) as opposed to manual placement (OCT manual, FFPE manual). We hope that the revised approach to naming the different sample types mentioned above makes this easier to follow.

- The venn diagrams in Figure 2e and 2f are misleading as visually they show a similar story, but numbers wise they are very different. Same for Figure 3d.

**Response:** We thank the reviewer for bringing this to our attention. The venn diagrams in Figures 2e, 2f, 3d and Supplementary Figure S4b have been revised with scaled circles to accurately reflect the degree of overlap.

**Reviewer #2:**

Du et al. present SpatialBench, a multi-sample, multi-protocol 10X Genomics Visium dataset that can be used as a practical guide to design and analyze spatial transcriptomics experiments.

The manuscript is well-written and presents a comprehensive dataset that convincingly compares different versions of the 10X Genomics Visium method applied to malaria-infected mouse spleen samples.

The first part of the manuscript is very useful as it systematically compares CytAssist versus manual Visium as well as FFPE versus fresh frozen samples. These results can serve as a guideline for researchers needing to decide on which protocol to use for their experiments.

In the second part of the manuscript, the authors showcase an R/Bioconductor-based workflow representing a typical downstream analysis of multi-sample, multi-condition Visium data: they first show that their approach can distinguish sex-specific gene expression in control samples and then apply their pipeline to comparing knock-out versus control infected samples, showing a good concordance with a previously published bulk RNA-seq experiment.

Overall, I enjoyed the paper and I believe that the SpatialBench dataset has the potential to be a valuable resource for the research community.

**Response:** We thank the reviewer for their supportive feedback on our work and are very glad to hear that they see value in the research

However, to live up to this expectation, the authors should address the following points.

1. To maximize the usability of the dataset for methods' benchmarking, I would strongly encourage the authors to package the data in a user-friendly format, such as an R or Bioconductor data package, as previously done by others with, e.g., the SpatialLIBD data (<https://bioconductor.org/packages/release/data/experiment/html/spatialLIBD.html)>. Ideally, interested researchers could load the data in standard format (e.g., SpatialExperiment or similar) with one line of code, as done in SpatialLIBD.

**Response:** We thank the reviewer for their feedback. We have deposited SpatialExperiment objects and metadata for all samples, which can be downloaded from our new zenodo repository [(https://zenodo.org/doi/10.5281/zenodo.12683146)](https://zenodo.org/doi/10.5281/zenodo.12683146) in the tibble object named

“all_samples.qs” inside SpatialBenchVisium.tar.gz (can be loaded in R with the command qs::qread(“all_samples.qs”)).

2. In the same spirit, I noticed that the data on GEO only include the "hires" and "lowres" versions of the H&E image from Visium. I suspect that the authors used a much higher resolution of the image when performing their QuPath analysis (sometimes referred to as "original resolution"). Was that the case or was the "hires" image used? If the former, it would be important for the reproducibility of the paper and for the importance of the resource for the authors to share (e.g., through FigShare or Zenodo) the original H&E images. This will be important for researchers wanting to benchmark methods that make use of imaging features in addition to gene expression for the analysis of Visium data.

**Response:** We thank the reviewer for bringing this to our attention. Higher resolution images were indeed used for the QuPath analysis. We have deposited the higher resolution tiff images for all samples in our newly created Zenodo repository that supports this data

analysis	[(https://zenodo.org/doi/10.5281/zenodo.12683146)](https://zenodo.org/doi/10.5281/zenodo.12683146)	in	the	file
SpatialBenchVisiumImages.tar.gz.

3. It would be useful to have a better global picture of the relative contribution of technical and biological variables to the overall variation observed in the data. For instance, are FFPE samples from different genotypes more or less similar to control samples assayed with FFPE or OCT techniques? One way of achieving this could be to look at the variance explained by each variable (e.g., sex, genotype, FFPE/OCT, CA/manual, ...). This can be done at the single-cell level, e.g., by looking at the variance explained by each principal component of the merged data and relating that to the above-mentioned variables, or perhaps more easily, at the pseudo-bulk level, by looking at multi-dimensional scaling plots color-coded by the different variables. An overall assessment of batch effects and what aspects of the protocols have a larger effect on separating batches would also be useful.

**Response:** We have added a pseudo-bulk MDS plot which shows the relationships between samples prepared and processed in different ways. This has been included as the new Supplementary Figure S3 and is repeated below. Briefly, this figure shows separation based on library preparation method (poly-A versus probe-based) explaining the most variation in dimension 1 and 2.

−0.5	0.0

**MDS2 (26.90%)**

−0.25

−0.50

0.50

0.25

0.00

544

709

174

168

167

709

708

460

173

709

713

experiment

FFPE manual (earlier) OCT manual (KOvsCTRL) OCT manual (WT) FFPE manual (later)

CytAssist

545

protocol

OCT manual FFPE manual OCT CA FFPE CA

463

710

462

713

709

713

708

0.5

**MDS1 (42.42%)**

**Supplementary Figure S3.** MDS plot of pseudo-bulk counts for all samples based on the 488 SVG genes detected across all wild-type samples (as shown in Figure 3c).

4. The authors did a great job at comparing the different protocols in terms of sample quality (using per-spot QC metrics), but it would be useful to look at the impact of the protocol in downstream results, e.g., clustering. One way to achieve this would be to look at cluster concordance across protocols and cluster stability within each protocol. For instance, the authors could perform cluster analysis for each protocol separately and then look at how the cell type distributions change between them; this analysis will likely be confounded with the biological differences between genotypes and the individual morphology of the samples, but it could give a rough idea of whether any strong bias is present in any of the methods. Alternatively, they could look at the concordance between marker gene lists identified downstream of clustering performed separately for each protocol.

**Response:** We thank the reviewer for their suggestion. Cluster analysis was indeed conducted on each protocol – we now include a new figure, Supplementary Figure S8 (see below), showing the cell type annotations for protocols not previously shown in Figure 3 (i.e. OCT manual (**S8a**), OCT CA (**S8b**) and FFPE manual (**S8c**)). In a comparison of cell type compositions across all protocols (**S8d**) we don’t observe any strong bias between different methods.

**Supplementary Figure S8.** Cell type annotation and composition across all protocols. (**a**) OCT manual samples with each spot annotated following deconvolution and marker gene expression analysis, using spatial clusters. (**b**) OCT CA samples with each spot annotated following deconvolution and marker gene expression analysis, using spatial clusters. (**c**) FFPE manual samples with each spot annotated following deconvolution and marker gene expression analysis, using spatial clusters. (**d**) The cell type composition of spatial clusters in each protocol, shown as percentages of the total spot count across all samples in the protocol.

5. The authors should improve Figure 1b to give a clearer overview of the experimental design. It took me a lot of back and forth between the main text, the methods section, and the supplementary tables to clearly understand how the experiment was designed. In my opinion the "Sample preparation, library construction, sequencing, data analysis" part of Figure 1b is unnecessary and remove it would make room for a more detailed version of the "Experimental design" table, perhaps merging information from Figure 1b and Supplementary Table S1. It would also be useful to clarify upfront what "KO" and "CTL" mean in terms of their genotype.

**Response:** We apologise for the confusion that our earlier figure caused and have revised if to improve clarity. The "Sample preparation, library construction, sequencing, data analysis" section has been simplified and made smaller to allow additional space for the experimental design panel in **Figure 1b** (see below), in a similar format to Supplementary Table S1. We explain the *T-bet* KO and CTL genotypes further in the Introduction and Results.

**Figure 1 (b)** 13 samples were captured over 4 10x Genomics Visium OCT slides and 3 FFPE slides, and sequenced over 5 runs on an Illumina NextSeq 2000. Samples are categorised by sex, genotype, tissue preparation protocol, library construction protocol, and tissue placement. A matching scRNA-seq sample of 3 mouse spleens was captured over 1 gel bead-in emulsion (GEM) well, and gene expression and hashtag oligos (HTOs) were sequenced over 1 Illumina run.

Minor issues:

- I found it confusing to refer to the individual experiments as "Experiment 1", "Experiment 2", etc. Could the authors use more descriptive names? Such as "FFPE manual", "FFPE CA", etc?

**Response:** Again, we apologise for the confusion. We have removed experiment numbers and now refer to samples based on their handling method (OCT manual, FFPE manual, OCT

CA, FFPE CA) as suggested, and to individual sample batches more descriptively when required. We have also edited the Supplementary Tables S1 and S2 in accordance.

**Reviewer #3:**

This benchmarking study by Du, Wang, Law, Ammann-Zalcenstein et al. has potential to aid researchers in selecting the appropriate 10x Visium-based approach for Spatial Transcriptomics. The authors make a rather extensive comparison of four approaches used on the same tissue or same tissue type. The experimental workflow is easy to follow, and the results are well-described in both text and figures. Unfortunately, I am skeptical about the comparison due to some major concerns stated below.

The presented data analysis workflow and results do not pose any significant advancements, which in my opinion is acceptable if the aim of the manuscript is to benchmark the wet lab protocols. However, unless the benchmarking part can be heavily revised, I suggest that the authors change the focus of the study and make it more biology-oriented. Surely, there are findings to be mined from this rich dataset.

I have used the following abbreviations in my comments: page X, column Y, row Z -> pXcYrZ. Other abbreviations are equivalent to those used in the manuscripts.

**Response:** Thank you for the comment. We have revised the benchmarking component of the manuscript, but now also highlight novel information that this approach revealed and could not be inferred from our earlier bulk RNA-seq approaches on sorted germinal centre B cells of malaria-infected mice (Ly *et a!.* 2019 Cell Reports). For example, our experiments using conditional deletion of transcription factor *T-bet* in the B cell compartment, suggested that in addition to influencing B cell activity, the lack to this transcription factor has an important downstream effect on T cells, with 106 genes differentially expressed in the spatially identified T cell cluster in spleens of malaria infected mice. Pathway enrichment analysis revealed enrichment of terms involved in antigen receptor-mediated signaling and cytoplasmic translation significantly altered in T cells by the absence of *T-bet* in B cells (Supplementary Figure S14), suggesting indirect effect influencing T cell function resulting from cognate interactions between wild-type T cells and *T-bet*-deficient B cells.

Our approach was also able to identify pathways in GC B cells influenced by the absence of T-bet that were not previously seen applying RNA-sequencing of sorted cells (Ly et al 2019). It has been previously found that ubiquitination prevents premature exit of antigen-experienced B cells from the germinal centre reaction (Li *et a!.* 2018 Immunity, 48: 530-541). Our previous findings demonstrated a critical role for *T-bet* promoting the induction of B cells with enhanced mutation in their Ig genes and increased affinity for antigen (Ly *et a!.* 2019 Cell Reports). Our new findings here show several terms involved in ubiquitination significantly downregulated by the absence of *T-bet* in GC B cells, suggesting that this

transcription factor might contribute to the enhanced ubiquitination activity required to allow the development of high affinity B cells in the germinal centre reaction.

These new results (highlighted in red) appear at the end of the Results section and in the Discussion.

Major comments:

1)	Comparing UMIs.

UMIs per spot is used as sensitivity (and performance) measure throughout the manuscript. However, there may be an important difference in what a UMI represents in 10x's poly-A versus probe-based approaches. In the former, a UMI always equals one captured transcript whereas in probe-based protocols there are up to three probe pairs per target transcript, all with different UMIs. Resultingly, the same number of original transcripts profiled with the two approaches may lead to three times more UMIs in the FFPE and CA experiments compared to FF (without CA). The number of probes (and UMIs) per target are different for the different versions and the issue may in any case be resolved bioinformatically. The fact that this is not mentioned (nor what probe-set was used) makes me doubt the entire comparison.

**Response:** We thank the reviewer for providing these details. We mentioned the probe set in the Results but agree that this was not clear. The probe set used is now explicitly stated in the Methods. Our dataset was pre-processed with the Visium Mouse Transcriptome Probe Set v1.0, which was designed with one probe-pair per transcript, in contrast to Probe Set v2.0 which, as the reviewer alluded to, contains on average three probe-pairs per transcript (refer to page 5 of this 10x Genomics Technical Note: [https://www.10xgenomics.com/support/cytassist-spatial-gene-](https://www.10xgenomics.com/support/cytassist-spatial-gene-expression/documentation/steps/sequencing/visium-cyt-assist-spatial-gene-expression-for-ffpe-robust-data-analysis-with-minimal-impact-of-genomic-dna)

[expression/documentation/steps/sequencing/visium-cyt-assist-spatial-gene-expression-for-ffpe-robust-data-analysis-with-minimal-impact-of-genomic-dna.](https://www.10xgenomics.com/support/cytassist-spatial-gene-expression/documentation/steps/sequencing/visium-cyt-assist-spatial-gene-expression-for-ffpe-robust-data-analysis-with-minimal-impact-of-genomic-dna) We can therefore expect the number of UMIs per spot to be comparable across poly-A- and probe-based approaches.

The relevant text, with edits highlighted in red are below:

1. Samples had a mean of 39,616,270 valid UMI counts, that is, UMIs covered by tissue. As we used a probe set designed with one probe-pair per transcript (30), the number of UMIs per spot were expected to be comparable across poly-A- and probe-based approaches. Across each experiment...

2. Pre-processing: Visium. Sequences were aligned to the mm10 transcriptome with the Visium Mouse Transcriptome Probe Set v1.0 and default parameter settings, and gene expression counts were obtained using Space Ranger *count*.

2) Tissue freezing and RNA quality.

The freezing protocol can affect RNA quality, and I find it unfortunate that the authors do not give any details on the freezing strategy. Also, I cannot find any measurement of the RNA quality in the fresh-frozen samples, which can strongly impact the performance of the poly-A capture protocol. RIN values should be provided for the FF samples and DV50 for FFPE.

**Response:** We apologise for not providing these details. A PrestoCHILL instrument was used to freeze OCT samples and we now include RIN values for OCT samples and DV200 scores for FFPE samples in Supplementary Table S2. The relevant text from the Methods, with edits highlighted in red is below:

**Sample preparation and library construction.** Twelve days post-infection, mice were euthanised and spleens were fixed in 10% v/v formalin and Paraffin-Embedded (FFPE) or embedded in Optimal Cutting Temperature compound (OCT) prior to freezing using a PrestoCHILL instrument. The DV200 scores or RIN values were checked for every FFPE or OCT block respectively. All FFPE blocks had a DV200 >80% and all OCT blocks had a RIN of >8.5.

3) Sub-optimal permeabilization.

The authors acknowledge that permeabilization in the FF experiments may have been suboptimal. Tissue optimization is undoubtedly a challenge when working with the FF material, but for a benchmarking study I am concerned that a suboptimal permeabilization will skew the whole comparison.

**Response:** We thank the reviewer for raising this point. We suggested sub-optimal permeabilisation as a potential issue. However, permeabilisation time was based on the results from the Visium Spatial Tissue Optimization kit which was performed under the guidance of 10x, and closely resembled the time obtained in-house by them. Perhaps it is then a reflection on the protocol itself. We have reworded the results and discussion to clarify this and added an image of the permeabilisation time course results as Supplementary Figure S1 4.

The relevant text, with major edits highlighted in red:

- An edge bias was also apparent, characterised by higher UMI counts along the

edges of the OCT samples. This	raises the possibility of under-permeabilisation

following	tissue optimisation, before library preparation and sequencing.

- In these poly-A-based samples, we also detected a bias showing higher UMI counts along tissue edges. A possible explanation could be sub-optimal permeabilisation conditions in our experiments, which can impact sensitivity and spatial resolution. Unlike FFPE manual and CytAssist workflows which use standard conditions, OCT manual sample preparation involves tissue optimisation experiments to determine optimal permeabilisation conditions for tissues before library sequencing. Yet our permeabilisation time was established following optimisation performed under 10x Genomics' guidance, closely aligning with their in-house results. Overall, our results may therefore indicate inherent limitations with the poly-A-based protocol.

**Supplementary Figure S14**. Permeabilisation time course results obtained from the Visium Spatial Gene Expression Tissue Optimization kit. Mouse E12.5 embryo was used as a positive control, with a permeabilisation time of 30 mins. Spleen tissue sections are labelled with increasing permeabilisation times, from 10 mins up to 60 mins.

4)	Sequencing depth.

"Samples were sequenced according to 10x's guidelines". This is indeed a good starting point, but samples differ in complexity and RNA quality. To really assess the performance

of the tissue handling protocols, samples should be sequenced to saturation. At the very least, an estimation of sequencing saturation per sample should be provided.

**Response:** We thank the reviewer for this suggestion. We have added the sequencing saturation metric from the Space Ranger output for each sample to **Supplementary Table S2**. We observed that all samples surpassed the minimum total sequencing depth recommended by 10x, calculated using tissue coverage multiplied by the recommended minimum of read pairs per spot covered with tissue (50,000 for OCT and 25,000 for FFPE and CytAssist). We describe this in the results and discussion.

The relevant text, with major edits highlighted in red:

- Across each experiment, the poly-A-based OCT manual samples had a mean of 23,642,694 valid UMI counts with sequencing saturation ranging from 0.70 to 0.77. For probe-based samples (FFPE manual and CA) the valid UMI count mean was higher at 41,630,649 with notably lower sequencing saturations of 0.24-0.53, except for FFPE CA sample 713 (0.79).
- This contrasts with probe-based capture methods, where probe sets exhibit higher specificity to a pre-defined number of known sequences, which may explain the lower average sequencing saturation, if the library complexity has been restricted.

5)	Adaptive immune receptor variable genes.

I assume IGHV..., IGLV..., TRBV... etc. were among the genes that were exclusively detected in OCT (poly-A-based) samples, as these are not included in the probe set. The authors should comment on whether these genes were detected, and make sure to exclude them prior to clustering as the can drive cluster formation in a way that is not biologically meaningful. Depending on their abundance in the data, there is however the potential to use them for VDJ analysis, which should also be commented on.

**Response:** Yes, IGLV genes were detected in OCT samples, though they were overall quite lowly ranked among the spatially variable genes (SVGs) used for spatial clustering (IGLV1: 97, IGLV3: 510). We agree with the suggestion to exclude such genes before clustering and for this reason had filtered out highly abundant haemoglobin and immunoglobulin genes from the top SVGs before performing spatial clustering. Notably, OCT samples had more of such genes among the top ranks than other sample groups. When exploring further exclusion of IGLV and more stringent filtering of immunoglobulin genes, there were no major changes to the spatial clustering.

Minor comments:

6) Definition of detected genes.

There is a significant difference in how many genes were detected depending on cut-off ( ≥3 UMIs in at least 10% or 1% of spots under tissue). How was the cut-off selected?

**Response:** We selected two cut-offs, one (1%) akin to the default settings recommended for the 10x Visium platform by the authors of one of the packages used for filtering genes. The other (10%) was more stringent and chosen after exploring several cut-offs to allow us to further investigate the differences in more highly expressed genes between protocols.

7) Detected genes in OCT samples.

On p4c2r2 the authors state that across the samples, 1,636 genes were detected in OCT samples, with a reference to Fig2d. This panel however shows only WT samples. From Fig2d, I end up with 1,720 genes when summarizing, same as in Fig2f. Please clarify, are 1,636 the mean number across all samples? Also, please clarify what samples are shown in Fig2e-f (I'm guessing wt samples, same as in Fig2d?).

**Response:** We apologise for the confusion and thank the reviewer for alerting us to this discrepancy. In Figure 2d and f, there are indeed 1,720 detected genes for OCT samples. 1,636 corresponded to an older version of the figure which had been corrected, but we had neglected to correct the relevant text. Figure 2d-f represent all samples, not just wildtype. The figure caption has been edited to clarify this.

8) Overlap of genes in probe-based samples.

"Similar numbers in the overlap of probe-based samples were observed for both thresholds, 4,000 genes." (p3c2r29) From Fig2d and figS2 my understanding is that the overlap is 5,142 and 8,343 respectively. What is the number 4,000 derived from?

**Response:** Thanks for the question. Our intention here was to highlight the similar numbers of genes (~4,000) found in all 3 probe-based protocols (FFPE CA, FFPE, and OCT CA) at both thresholds. For Figure 2d showing the 10% threshold, this corresponded to only the first, largest peak of 3,708 genes. For Figure S2, this was the second largest peak of 3,775 genes. This contrasted with the peak overlapping of all 4 protocols which differed greatly in number of detected genes between thresholds. We have rephrased this sentence to improve clarity and include more accurate gene numbers.

The relevant text, with edits highlighted in red:

At a more lenient threshold of detected genes being defined as genes with a count of 3 or more in at least 1% of spots (Supplementary Figure S2), the largest overlap in detected genes instead includes all 4 sample types, with more than 3 times the number of genes than at the stricter threshold. This implies that most of the original overlapping genes between poly-A­based and probe-based samples were very lowly expressed under the poly-A protocol. On the contrary, at both thresholds, the peak overlapping all probe-based samples showed similar numbers of genes (3,708 at 10%, 3,775 at 1%).

9) "We have created SpatialBench, a unique multi-sample spatial benchmarking dataset while profiles the mouse spleen as a reference tissue, generated using 10x Genomics' Visium spatial technology." (p10c1r23-26). I do not understand this phrase.

**Response:** Apologies for the confusion. We intended to say “which profiles the mouse spleen”. This has now been fixed.

10) Typo: P4c2r58 "along" -> "alone"
    **Response:** Thank you, this has been fixed.
11) P8c1r61: duplication of "the"
    **Response:** Thank you, this has been fixed.
12) "In these samples, we also detected a bias showing higher UMI counts along tissue edges." (p10c1r54-54) "These samples" refer to OCT (poly-A based) samples, yes? Please clarify to avoid confusion.

**Response:** Yes, apologies for the confusion. We refer to this more clearly now as “these poly-A-based samples”.

13) Fig 3g seems to be referenced prior to Fig 3f in the text. It would be easier to follow if figures followed the same order as r

**Response:** Thanks for pointing this out - the paragraphs in this section (Cell type deconvolution) have been re-ordered to accurately reflect the order in which the panels in Figure 3 appear.

**Second round of review**

**Reviewer 1**

The authors have done a fantastic job addressing all of my comments and questions. I have no other concerns. I applaud the authors for the great work and look forward to sharing this work with colleagues who are interested in better understanding the advantages / disadvantages of these technologies.

**Reviewer 2**

The authors addressed all my comments. Congratulations on a nice study!

**Reviewer 3**

I enjoyed the revised version of the manuscript. The added details and transparency enhance the reader’s ability to assess the performance of the different protocols, and the workflow is now clearer. As a result, the benchmarking study has improved significantly and I believe it is a valuable resource for the community.

However, I am slightly confused about the results presented at the end of the Results section. In the rebuttal letter, the authors refer to Figure S14 for these results, but this figure shows the permeabilization time course. Has the intended figure been omitted during resubmission? Please locate and include it in the final version.

Below are some comments on my previous concerns:

1. I appreciate that the authors looked into the numbers of probe pairs per target and addressed this in the manuscript.
2. Similarly, I’m glad to see that sample quality was satisfactory. However, I could not locate the per-sample information in Table S2, as referenced in the rebuttal letter.
3. I agree with the authors that the tissue optimization assay is a challenging step and may be an inherent limitation of the poly-A-based protocol. Including the permeabilization scans makes the reasoning easier to follow.
4. It was interesting to know the estimated sequencing saturation, although I do not quite agree with the reasoning on page 9 row 55:

“This contrasts with probe-based capture methods, where probe sets exhibit higher specificity to a pre-defined number of known sequences, which may explain the lower average sequencing saturation, if the library complexity has been restricted.”

A lower average sequencing saturation suggests higher library complexity. To me, the results indicate that the probe-based methods captured more transcripts (regardless of number of genes), required fewer PCR cycles and kept a higher library complexity.

**Authors Response**

**Point-by-point responses to the reviewers’ comments:**

Reviewer #3: I enjoyed the revised version of the manuscript. The added details and transparency enhance the reader’s ability to assess the performance of the different protocols, and the workflow is now clearer. As a result, the benchmarking study has improved significantly and I believe it is a valuable resource for the community.

However, I am slightly confused about the results presented at the end of the Results section. In the rebuttal letter, the authors refer to Figure S14 for these results, but this figure shows the permeabilization time course. Has the intended figure been omitted during resubmission? Please locate and include it in the final version.

**Response:** Thank you for pointing out the missing supplementary figure, which we accidentally left out of the revised manuscript (very sorry for that!) We have included this and numbered it correctly as Fig. S14 (copied below) in the revised version, with the permeabilization time course shown as Fig. S15.

**Fig. S14.** *Dot plot of KO vs CTL T cell cluster GO term enrichment analysis*. Dot plot showing the top-ranked GO biological process terms enriched in the T cell cluster for the KO vs CTL comparison. The top 10 most significant terms are plotted, coloured by the adjusted *p*value and sized by the number of differentially expressed genes in the term. The x-axis represents the ratio of genes in the term over the total number of differentially expressed genes.

Below are some comments on my previous concerns:

1. I appreciate that the authors looked into the numbers of probe pairs per target and addressed this in the manuscript.
2. Similarly, I’m glad to see that sample quality was satisfactory. However, I could not locate the per-sample information in Table S2, as referenced in the rebuttal letter.

Response: Apologies again (I think I uploaded an older version of the Supp Figures file by mistake). We have added the RIN values for OCT samples and DV200 scores for FFPE samples to Additional file 2: Table S2 and copied them below.

Table S2 (copy of first 6 columns)

3. I agree with the authors that the tissue optimization assay is a challenging step and may be an inherent limitation of the poly-A-based protocol. Including the permeabilization scans makes the reasoning easier to follow.
4. It was interesting to know the estimated sequencing saturation, although I do not quite agree with the reasoning on page 9 row 55:

“This contrasts with probe-based capture methods, where probe sets exhibit higher specificity to a pre-defined number of known sequences, which may explain the lower average sequencing saturation, if the library complexity has been restricted.”

A lower average sequencing saturation suggests higher library complexity. To me, the results indicate that the probe-based methods captured more transcripts (regardless of number of genes), required fewer PCR cycles and kept a higher library complexity.

Response: Thank you for bringing this to our attention. We agree with your comment and have revised the sentence to reflect this reasoning (see below, edit highlighted in red):

“This contrasts with probe-based capture methods, where probe sets are highly specific to a predefined set of known sequences. This specificity, combined with workflow modifications, may explain the lower average sequencing saturation observed compared to the less efficient poly-A based capture of full-length transcripts.”
